# 1,4-Bis[4-(*tert*-butyl­sulfan­yl)phen­yl]buta-1,3-diyne

**DOI:** 10.1107/S1600536812017710

**Published:** 2012-04-28

**Authors:** Nadine Seidel, Sebastian Förster, Wilhelm Seichter, Edwin Weber

**Affiliations:** a Institut für Organische Chemie, TU Bergakademie Freiberg, Leipziger Strasse 29, D-09596 Freiberg/Sachsen, Germany

## Abstract

The asymmetric unit of the title compound, C_24_H_26_S_2_, consits of one half-mol­ecule, which is located on a center of inversion. The two benzene rings are exactly coplanar while the *tert*-butyl group is oriented nearly perpendicular to the ring plane [C—S—C—C = −81.14 (11)°].

## Related literature

For background to this work, see: Pearson & Tour (1997 ▶); Kergueris *et al.* (1999 ▶). For related structures, see: Kergueris *et al.* (1999 ▶); Mayor *et al.* (2003 ▶). For the synthetic procedure, see: van Dijk *et al.* (2006 ▶). For the unsubstituted 1,4-diphenyl­buta-1,3-diyne, see: Hori *et al.* (1987 ▶).
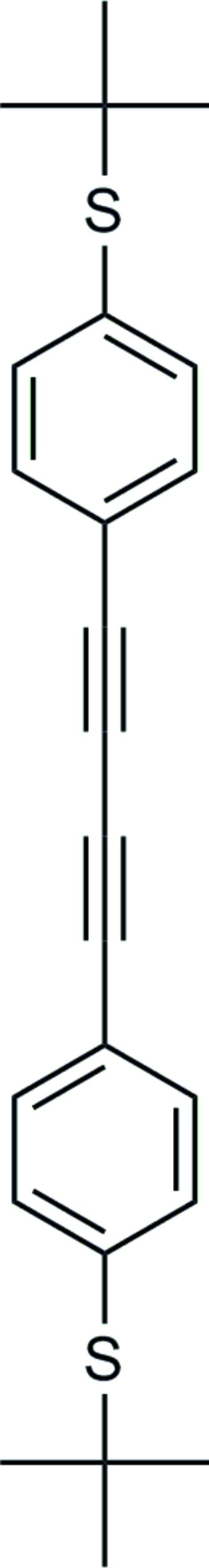


## Experimental

### 

#### Crystal data

C_24_H_26_S_2_
*M* *_r_* = 378.57
Monoclinic, 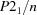

*a* = 13.6286 (5) Å
*b* = 6.4269 (2) Å
*c* = 14.1290 (5) Å
β = 116.937 (2)°
*V* = 1103.29 (7) Å^3^
*Z* = 2
Mo *K*α radiation
μ = 0.25 mm^−1^
*T* = 153 K
0.53 × 0.20 × 0.05 mm

#### Data collection

Bruker APEXII CCD area-detector diffractometer
Absorption correction: multi-scan (*SADABS*; Sheldrick, 2004 ▶) *T*_min_ = 0.881, *T*_max_ = 0.989
11825 measured reflections
3028 independent reflections
2450 reflections with *I* > 2σ(*I*)
*R*_int_ = 0.025

#### Refinement

*R*[*F* ^2^ > 2σ(*F* ^2^)] = 0.034
*wR*(*F* ^2^) = 0.091
*S* = 1.04
3028 reflections
121 parameters
H-atom parameters constrained
Δρ_max_ = 0.35 e Å^−3^
Δρ_min_ = −0.20 e Å^−3^

### 

Data collection: *APEX2* (Bruker, 2007 ▶); cell refinement: *SAINT* (Bruker, 2007 ▶); data reduction: *SAINT*; program(s) used to solve structure: *SHELXS97* (Sheldrick, 2008 ▶); program(s) used to refine structure: *SHELXL97* (Sheldrick, 2008 ▶); molecular graphics: *ORTEP-3* (Farrugia, 1997 ▶); software used to prepare material for publication: *SHELXTL* (Sheldrick, 2008 ▶).

## Supplementary Material

Crystal structure: contains datablock(s) I, global. DOI: 10.1107/S1600536812017710/nc2274sup1.cif

Structure factors: contains datablock(s) I. DOI: 10.1107/S1600536812017710/nc2274Isup2.hkl

Supplementary material file. DOI: 10.1107/S1600536812017710/nc2274Isup3.cml

Additional supplementary materials:  crystallographic information; 3D view; checkCIF report

## Figures and Tables

**Fig. 1.**
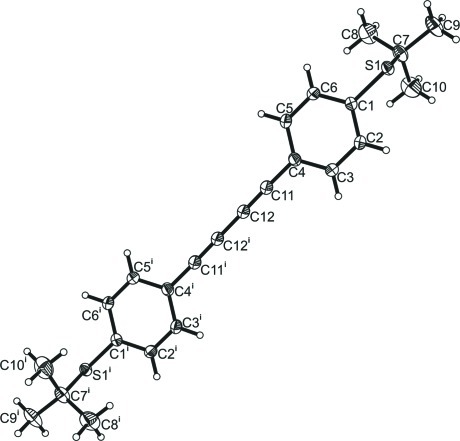
Perspective view of the title compound with labelling showing 50% probability displacement ellipsoids for the non-H atoms. Symmetry code: i = -x+2, -y, -z+2.

## supplementary crystallographic information

### Comment

Rod-type oligo(phenyleneethynylene)s are important representatives of conjugated molecular wires (Pearson *et al.*, 1997). Molecular rods consisting protected terminal thiol anchor groups have been embedded as passive elements in Molecular Break-Junctions (Kergueris *et al.*, 1999). As a part of our ongoing project on the synthesis of corresponding structures, the title compound was obtained as a by-product and was identified by single-crystal X-ray diffraction. It crystallizes with one half of the molecule in the unit cell, *i.e.* the molecule adopts inversion symmetry. The molecule features an almost planar geometry except for the *tert*-butyl groups which are slightly twisted out of the plane (C(7)—S(1)—C(1)—C(2) 81.15 (14) °, C(1)—S(1)—C(7)—C(10) -67.57 (12) °). Compared with the unsubstituted 1,4-diphenylbuta-1,3-diyne (Hori *et al.*, 1987), the position of atoms shows marginal differences. Bond distances of the ring are in the range 1.38–1.40 Å, the shortest being C(5)—C(6)=1.383 (19) Å. The angles in the ring are between 119.01 (12) and 120.47 (13) °. The C—S distance is in good agreement with the literature data [S(1)—C(1) 1.7747 (14) Å, S(1)—C(7) 1.8514 (17) Å] (Mayor *et al.*, 2003).

### Experimental

The title compound has been obtained as a by-product during attempted Sonogashira cross coupling reaction of *tert*-butyl-(4-ethynylphenyl)sulfane with 2,6-dibromoanthra-9,10-quinone in diisopropylamine. For the synthetic procedure, see: van Dijk *et al.*, (2006). The plate shaped crystals are colourless and stable in the air.

### Refinement

H atoms were positioned geometrically and allowed to ride on their respective parent atoms, with C—H = 0.95 Å.

### Crystal data

**Table:**

C_24_H_26_S_2_	*F*(000) = 404
*M**_r_* = 378.57	*D*_x_ = 1.140 Mg m^−^^3^
Monoclinic, *P*2_1_/*n*	Mo *K*α radiation, λ = 0.71073 Å
*a* = 13.6286 (5) Å	Cell parameters from 4805 reflections
*b* = 6.4269 (2) Å	θ = 2.8–30.5°
*c* = 14.1290 (5) Å	µ = 0.25 mm^−^^1^
β = 116.937 (2)°	*T* = 153 K
*V* = 1103.29 (7) Å^3^	Plate, colourless
*Z* = 2	0.53 × 0.20 × 0.05 mm

### Data collection

**Table:**

Bruker APEXII CCD area-detector diffractometer	3028 independent reflections
Radiation source: fine-focus sealed tube	2450 reflections with *I* > 2σ(*I*)
Graphite monochromator	*R*_int_ = 0.025
phi and ω scans	θ_max_ = 29.4°, θ_min_ = 2.8°
Absorption correction: multi-scan (*SADABS*; Sheldrick, 2004)	*h* = −17→18
*T*_min_ = 0.881, *T*_max_ = 0.989	*k* = −8→8
11825 measured reflections	*l* = −19→17

### Refinement

**Table:**

Refinement on *F*^2^	Primary atom site location: structure-invariant direct methods
Least-squares matrix: full	Secondary atom site location: difference Fourier map
*R*[*F*^2^ > 2σ(*F*^2^)] = 0.034	Hydrogen site location: inferred from neighbouring sites
*wR*(*F*^2^) = 0.091	H-atom parameters constrained
*S* = 1.04	*w* = 1/[σ^2^(*F*_o_^2^) + (0.0415*P*)^2^ + 0.3498*P*] where *P* = (*F*_o_^2^ + 2*F*_c_^2^)/3
3028 reflections	(Δ/σ)_max_ < 0.001
121 parameters	Δρ_max_ = 0.35 e Å^−^^3^
0 restraints	Δρ_min_ = −0.20 e Å^−^^3^

### Special details

**Table:**

Geometry. All e.s.d.'s (except the e.s.d. in the dihedral angle between two l.s. planes) are estimated using the full covariance matrix. The cell e.s.d.'s are taken into account individually in the estimation of e.s.d.'s in distances, angles and torsion angles; correlations between e.s.d.'s in cell parameters are only used when they are defined by crystal symmetry. An approximate (isotropic) treatment of cell e.s.d.'s is used for estimating e.s.d.'s involving l.s. planes.
Refinement. Refinement of *F*^2^ against ALL reflections. The weighted *R*-factor *wR* and goodness of fit *S* are based on *F*^2^, conventional *R*-factors *R* are based on *F*, with *F* set to zero for negative *F*^2^. The threshold expression of *F*^2^ > σ(*F*^2^) is used only for calculating *R*-factors(gt) *etc*. and is not relevant to the choice of reflections for refinement. *R*-factors based on *F*^2^ are statistically about twice as large as those based on *F*, and *R*- factors based on ALL data will be even larger.

### Fractional atomic coordinates and isotropic or equivalent isotropic displacement parameters (Å^2^)

**Table:**

	*x*	*y*	*z*	*U*_iso_*/*U*_eq_	
S1	0.93054 (2)	0.80456 (5)	0.55276 (2)	0.02231 (10)	
C1	0.93335 (10)	0.62309 (18)	0.64857 (9)	0.0206 (2)	
C2	0.90994 (11)	0.68270 (19)	0.73135 (10)	0.0250 (3)	
H2	0.8874	0.8214	0.7343	0.030*	
C3	0.91924 (11)	0.5417 (2)	0.80912 (10)	0.0261 (3)	
H3	0.9019	0.5832	0.8643	0.031*	
C4	0.95429 (10)	0.33764 (19)	0.80637 (10)	0.0234 (3)	
C5	0.97762 (11)	0.2782 (2)	0.72369 (10)	0.0257 (3)	
H5	1.0013	0.1402	0.7212	0.031*	
C6	0.96658 (11)	0.41878 (19)	0.64538 (10)	0.0247 (3)	
H6	0.9817	0.3760	0.5890	0.030*	
C7	0.78266 (11)	0.8255 (2)	0.45814 (11)	0.0308 (3)	
C8	0.73566 (15)	0.6125 (3)	0.41517 (14)	0.0523 (5)	
H8A	0.7786	0.5482	0.3831	0.078*	
H8B	0.6589	0.6271	0.3614	0.078*	
H8C	0.7391	0.5247	0.4733	0.078*	
C9	0.78203 (13)	0.9643 (3)	0.37009 (13)	0.0467 (4)	
H9A	0.7060	0.9860	0.3159	0.070*	
H9B	0.8241	0.8972	0.3378	0.070*	
H9C	0.8155	1.0989	0.4001	0.070*	
C10	0.72009 (13)	0.9298 (3)	0.51159 (14)	0.0480 (4)	
H10A	0.7237	0.8416	0.5697	0.072*	
H10B	0.6430	0.9492	0.4595	0.072*	
H10C	0.7534	1.0653	0.5399	0.072*	
C11	0.97122 (11)	0.1931 (2)	0.88969 (10)	0.0259 (3)	
C12	0.98954 (11)	0.0698 (2)	0.95990 (10)	0.0265 (3)	

### Atomic displacement parameters (Å^2^)

**Table:**

	*U*^11^	*U*^22^	*U*^33^	*U*^12^	*U*^13^	*U*^23^
S1	0.02344 (15)	0.02417 (15)	0.02180 (16)	0.00002 (11)	0.01242 (12)	0.00352 (11)
C1	0.0213 (5)	0.0224 (5)	0.0181 (6)	−0.0005 (4)	0.0090 (5)	0.0011 (4)
C2	0.0327 (7)	0.0210 (6)	0.0255 (6)	0.0017 (5)	0.0169 (5)	0.0000 (5)
C3	0.0347 (7)	0.0264 (6)	0.0222 (6)	0.0008 (5)	0.0173 (5)	−0.0011 (5)
C4	0.0267 (6)	0.0230 (6)	0.0205 (6)	−0.0010 (5)	0.0107 (5)	0.0013 (4)
C5	0.0330 (7)	0.0218 (6)	0.0237 (6)	0.0030 (5)	0.0141 (5)	−0.0002 (5)
C6	0.0307 (6)	0.0257 (6)	0.0212 (6)	0.0022 (5)	0.0148 (5)	−0.0005 (5)
C7	0.0244 (6)	0.0385 (7)	0.0259 (7)	0.0009 (5)	0.0083 (5)	0.0080 (6)
C8	0.0453 (9)	0.0535 (10)	0.0391 (9)	−0.0178 (8)	0.0026 (8)	−0.0021 (8)
C9	0.0375 (8)	0.0616 (11)	0.0362 (8)	0.0078 (7)	0.0125 (7)	0.0244 (8)
C10	0.0305 (8)	0.0661 (11)	0.0508 (10)	0.0153 (8)	0.0215 (7)	0.0150 (9)
C11	0.0321 (6)	0.0246 (6)	0.0235 (6)	0.0000 (5)	0.0147 (5)	−0.0011 (5)
C12	0.0340 (7)	0.0246 (6)	0.0246 (6)	0.0015 (5)	0.0167 (6)	−0.0007 (5)

### Geometric parameters (Å, º)

**Table:**

S1—C1	1.7740 (12)	C7—C9	1.528 (2)
S1—C7	1.8509 (13)	C7—C10	1.526 (2)
C1—C2	1.3980 (17)	C8—H8A	0.9800
C1—C6	1.3964 (17)	C8—H8B	0.9800
C2—C3	1.3852 (17)	C8—H8C	0.9800
C2—H2	0.9500	C9—H9A	0.9800
C3—C4	1.4022 (17)	C9—H9B	0.9800
C3—H3	0.9500	C9—H9C	0.9800
C4—C5	1.3957 (17)	C10—H10A	0.9800
C4—C11	1.4339 (17)	C10—H10B	0.9800
C5—C6	1.3828 (17)	C10—H10C	0.9800
C5—H5	0.9500	C11—C12	1.2042 (18)
C6—H6	0.9500	C12—C12^i^	1.370 (3)
C7—C8	1.517 (2)		
			
C1—S1—C7	103.83 (6)	C9—C7—S1	103.49 (10)
C2—C1—C6	119.01 (11)	C10—C7—S1	110.13 (10)
C2—C1—S1	121.60 (9)	C7—C8—H8A	109.5
C6—C1—S1	119.27 (9)	C7—C8—H8B	109.5
C3—C2—C1	120.72 (11)	H8A—C8—H8B	109.5
C3—C2—H2	119.6	C7—C8—H8C	109.5
C1—C2—H2	119.6	H8A—C8—H8C	109.5
C2—C3—C4	119.99 (12)	H8B—C8—H8C	109.5
C2—C3—H3	120.0	C7—C9—H9A	109.5
C4—C3—H3	120.0	C7—C9—H9B	109.5
C5—C4—C3	119.25 (11)	H9A—C9—H9B	109.5
C5—C4—C11	119.79 (11)	C7—C9—H9C	109.5
C3—C4—C11	120.89 (11)	H9A—C9—H9C	109.5
C6—C5—C4	120.49 (11)	H9B—C9—H9C	109.5
C6—C5—H5	119.8	C7—C10—H10A	109.5
C4—C5—H5	119.8	C7—C10—H10B	109.5
C5—C6—C1	120.52 (11)	H10A—C10—H10B	109.5
C5—C6—H6	119.7	C7—C10—H10C	109.5
C1—C6—H6	119.7	H10A—C10—H10C	109.5
C8—C7—C9	110.82 (14)	H10B—C10—H10C	109.5
C8—C7—C10	111.40 (14)	C12—C11—C4	177.35 (14)
C9—C7—C10	110.38 (13)	C11—C12—C12^i^	179.74 (18)
C8—C7—S1	110.35 (11)		
			
C7—S1—C1—C2	−81.14 (11)	C11—C4—C5—C6	−177.00 (12)
C7—S1—C1—C6	103.04 (11)	C4—C5—C6—C1	0.9 (2)
C6—C1—C2—C3	−0.12 (19)	C2—C1—C6—C5	−0.87 (19)
S1—C1—C2—C3	−175.95 (10)	S1—C1—C6—C5	175.06 (10)
C1—C2—C3—C4	1.1 (2)	C1—S1—C7—C8	−55.80 (12)
C2—C3—C4—C5	−1.11 (19)	C1—S1—C7—C9	−174.41 (11)
C2—C3—C4—C11	175.99 (12)	C1—S1—C7—C10	67.60 (12)
C3—C4—C5—C6	0.13 (19)		

Symmetry code: (i) −*x*+2, −*y*, −*z*+2.
